# Medial talar resection: how much remains stable?

**DOI:** 10.1007/s00068-022-01915-0

**Published:** 2022-02-23

**Authors:** Jennifer E. Hagen, Andrew K. Sands, Michael Swords, Stefan Rammelt, Nina Schmitz, Geoff Richards, Boyko Gueorguiev, Firas Souleiman

**Affiliations:** 1 AO Research Institute Davos, Davos, Switzerland; 2 Department of Orthopedics and Rehabilitation, Orthopedics University of Florida, Gainesville, FL USA; 3 New York Presbyterian-Lower Manhattan Hospital, New York, NY USA; 4 Michigan Orthopedic Center, Lansing, MI USA; 5 Department of Foot and Ankle Surgery, University Hospital Dresden, Dresden, Germany; 6 Department of Trauma, Hand, and Reconstructive Surgery, University Hospital Münster, Münster, Germany; 7 Department of Orthopedics, Trauma and Plastic Surgery, University Hospital of Leipzig, University of Leipzig, Leipzig, Germany

**Keywords:** weight-bearing, subtalar instability, talus fracture, talocalcaneal coalition, medial talar facet resection

## Abstract

**Purpose:**

Pathologies of the medial talus (e.g., fractures, tarsal coalitions) can lead to symptomatic problems such as pain and nonunion. Bony resection may be a good solution for both. It is unclear how much of the medial talus can be taken before the subtalar joint becomes unstable. The aim of this study was to evaluate the effect a limited resection of the medial talar facet and the anteromedial portion of the posterior talar facet has on subtalar stability.

**Methods:**

Eight fresh-frozen human cadaveric lower limbs were mounted in a frame for simulated weight-bearing. Computed tomography scans were obtained under 700 N single-legged stance loading, with the foot in neutral, 15° inversion, and 15° eversion positions. A sequential resection of 10, 20, and 30% of the medial facet and the anteromedial portion of the posterior talar facet to the calcaneus, based on the intact talus width, was performed. Measurements of subtalar vertical angulation, talar subluxation, coronal posterior facet angle and talocalcaneal (Kite) angle in the anteroposterior and lateral view were performed.

**Results:**

Gross clinical instability was not observed in any of the specimens. No significant differences were detected in the measurements between the resected and intact states (*P* ≥ 0.10) as well as among the resected states (*P* ≥ 0.11).

**Conclusion:**

In a biomechanical setting, resecting up to 30% of the medial facet and anteromedial portion of the posterior facet based on the intact talus width—does not result in any measurable instability of the subtalar joint in presence of intact ligamentous structures.

**Level of evidence:**

V.

## Background

Pathologies of the posterior medial body of the talus are rare, difficult to diagnose and pose an operative dilemma for surgeons. They can be difficult to approach, with risk to the posterior tibial neurovascular bundle, and are at risk for nonunion due to a complex blood supply [1–3]. The most common medial talar pathologies represent tarsal coalitions and fractures. Optimal treatment has not been determined. Some surgeons advocate for fixation and some for excision [4, 5].

*Talocalcaneal tarsal coalition* describes a congenital fusion of a portion of the subtalar joint, most often of the medial talar facet [6]. It occurs with a prevalence of 1–2% and could be symptomatic [7]. Often when conservative therapy fails, then operative procedures remain. Depending on the size of the coalition, fusions or resections can be performed [8]. The concern is that resection of large coalitions will create instability in the subtalar joint. Generally, collapse of this joint has significant impact on hindfoot misalignment and could lead to early arthritis in it and the neighbor joints.

*Talus fractures* occur with an incidence of less than 1% [9]. The reason is often a high impact of force [10]. Talar body fractures can occur in plantar flexion of the foot under axial loading [3]. In the event of fracture dislocation, rapid reduction and fixation should be performed to preserve perfusion of both the bone and soft tissues [3]. Despite open reduction with internal fixation to restore the joint surfaces, osteonecrosis and post-traumatic arthrosis are observed in the majority of cases [10]. When the diagnosis of medial talus fractures is delayed, resection may be appropriate, in particular in presence of medial joint facet comminution.

However, for both mentioned medial talus pathologies, it is unclear how much of the medial portion of the talus can be resected before the subtalar joint becomes unstable.

Therefore, the aim of this study was to evaluate the effect a limited resection of up to 30% of the medial facet and anteromedial portion of the posterior facet—based on the intact talus width has on subtalar joint stability. A combination of simulated weight-bearing with a computed tomography (CT) technology to assess cadaveric hindfoot alignment prior to and post resection of the medial talar facets was used. It was hypothesized that up to 30% of the medial facets of the talus based on the intact talus width can be resected without evidence for subtalar joint instability.

## Materials and methods

**Fig. 1:**
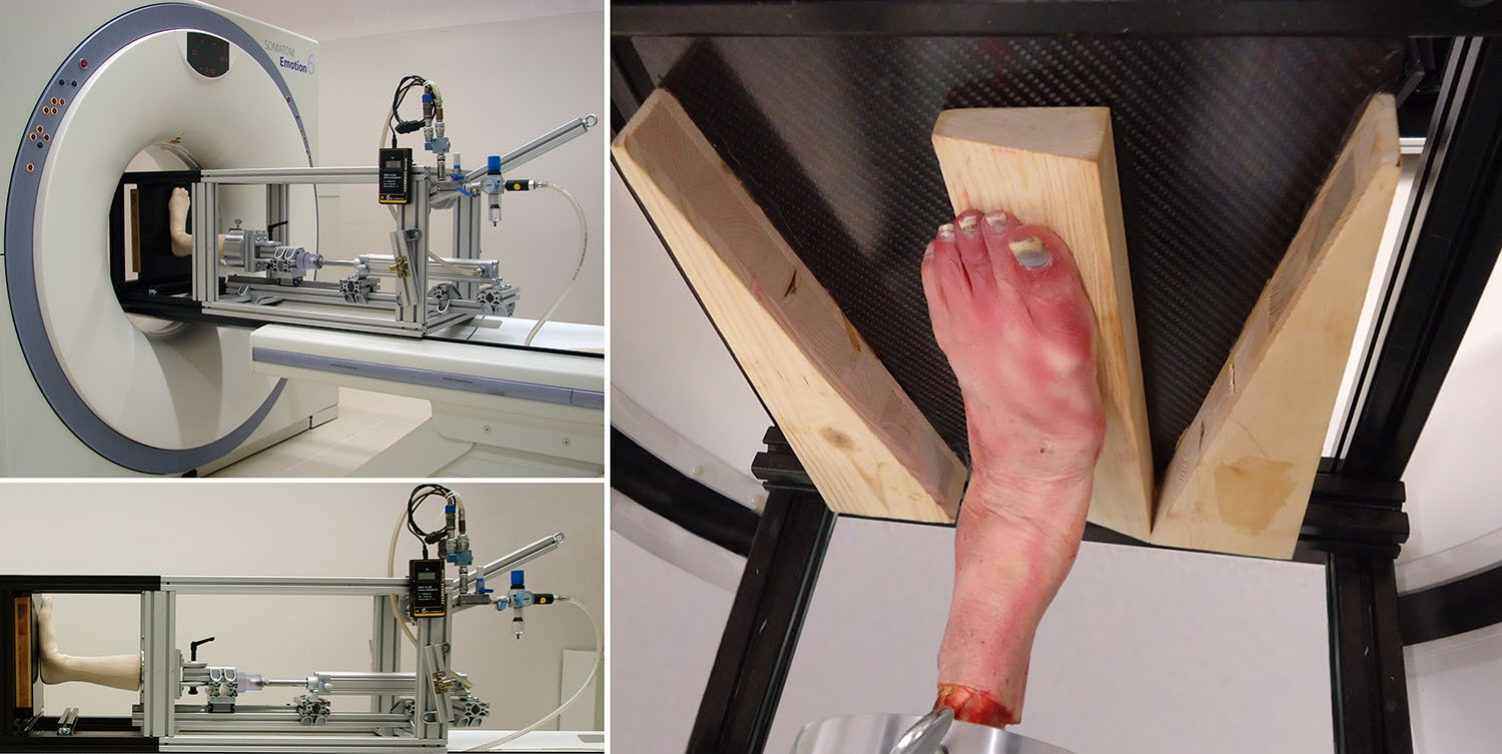
(Left) Custom-made loading frame with an artificial specimen mounted for CT scanning under weight-bearing. The distal end of the frame is made out of radiolucent composite material, the main part is made out of aluminum. A pneumatic cylinder connects to a compressed air system at the proximal end of the frame; (right) human cadaveric specimen mounted in the loading frame with foot oriented in 15° inversion by use of a 15° wooden wedge underneath the medial column of the foot

Eight fresh-frozen (− 20 °C) human cadaveric lower limbs were thawed to 3 °C, cut and embedded at the mid-tibia in polymethylmethacrylate (PMMA) cement (Beracryl, Suter Kunststoff AG, Jegenstorf, Switzerland) by means of a pre-fabricated cylindrical pot. Each specimen was mounted in a custom-made frame developed for CT scanning under simulated weight-bearing (Fig. 1).

**Fig. 2:**
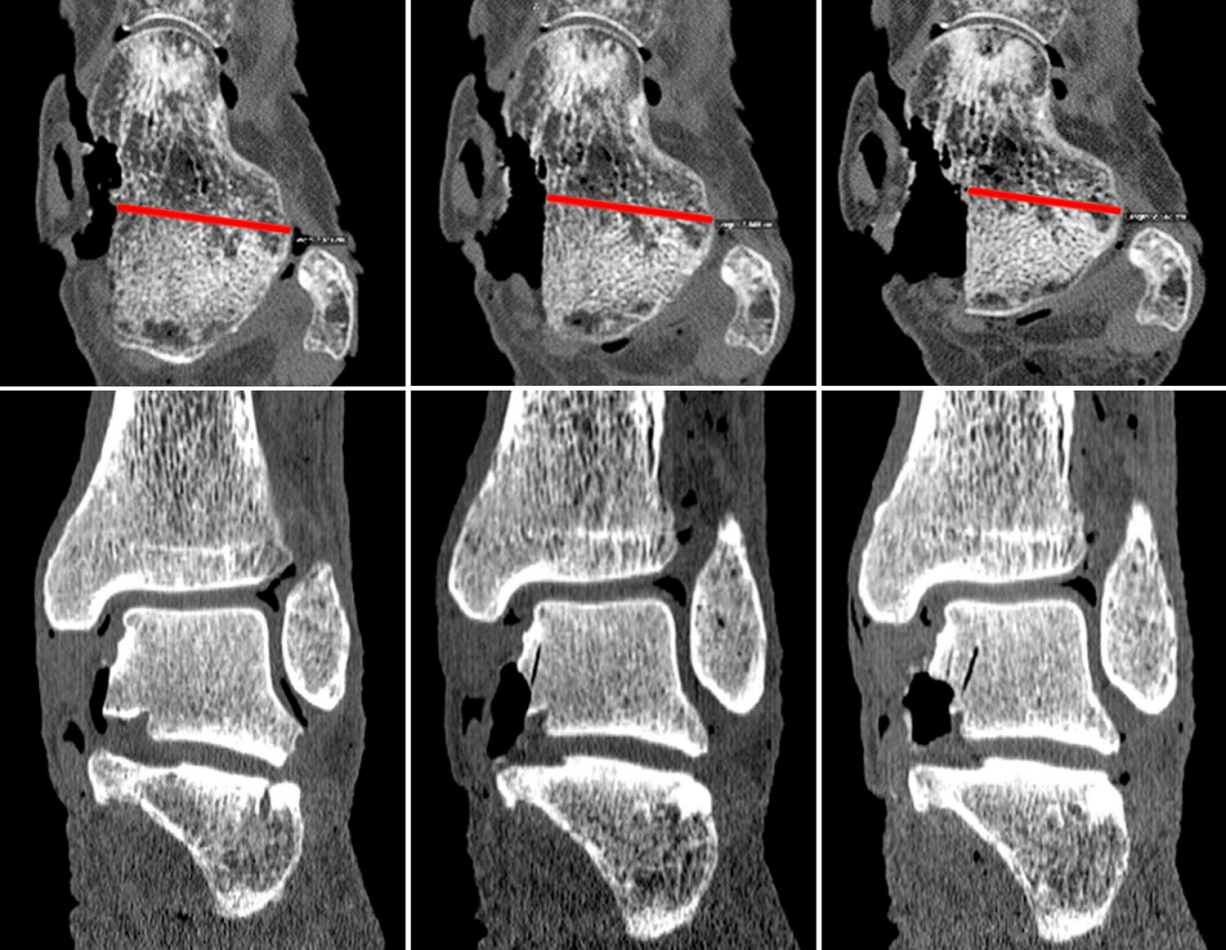
(Top) Axial CT views demonstrating 10, 20, and 30% resections of the medial talar facet and the anteromedial portion of the posterior talar facet; (bottom) coronal CT views demonstrating 10, 20 and 30% resections as described above

**Fig. 3:**
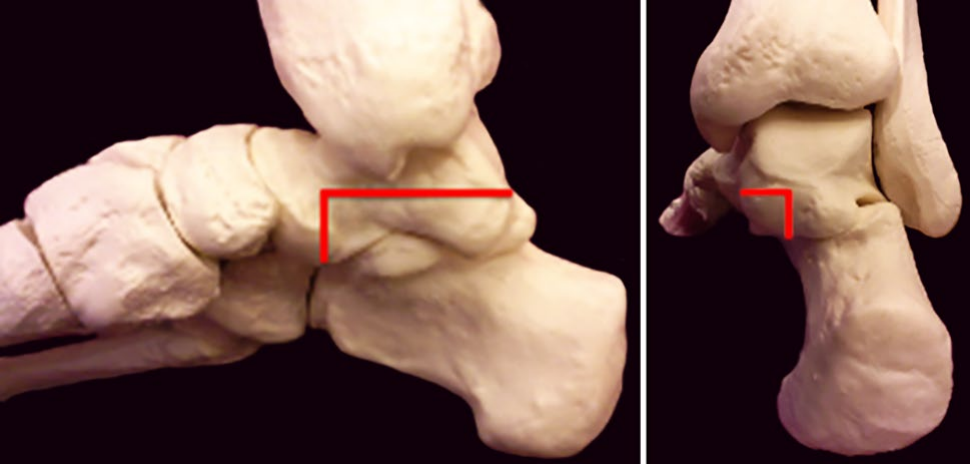
(Left) Medial and (right) posterior view of the hindfoot. Red lines demonstrate the resection site

The frame was designed to perform pneumatic static axial loading in the range from 0 to 750 N. The distal end of the frame was radiolucent to avoid scattering [11–13]. The specimens were positioned plantigrade on a carbon fiber plate. Wooden wedges were put underneath the medial or lateral column of the foot for inversion and eversion, respectively (Fig. 1). CT scans of 0.63 mm slice thickness were performed using a SOMATOM Emotion CT scanner (Siemens, Munich, Germany). Each specimen was scanned under 700 N single-legged stance loading while placing the foot in neutral, 15 degree inversion, or 15 degree eversion position [11–13]. The width of each intact talus perpendicular to its long axis at the level of the lateral process was measured using CT scanning. Via a combined anteromedial and posteromedial approach, the equivalence of 10, 20 and 30% based on this measured width were resected of the medial facet and the anteromedial portion of the posterior talar facet using a sharp osteotome and gauge (Fig. 2). Care was taken to avoid damage to the talar dome and head while preserving the superficial insertion of the deltoid and interosseous ligaments as visualized in Fig. 3. The deep deltoid insertion was detached. The specimens were rescanned under 700 N loading (single-legged stance) in all three foot positions following each resection.

**Fig. 4:**
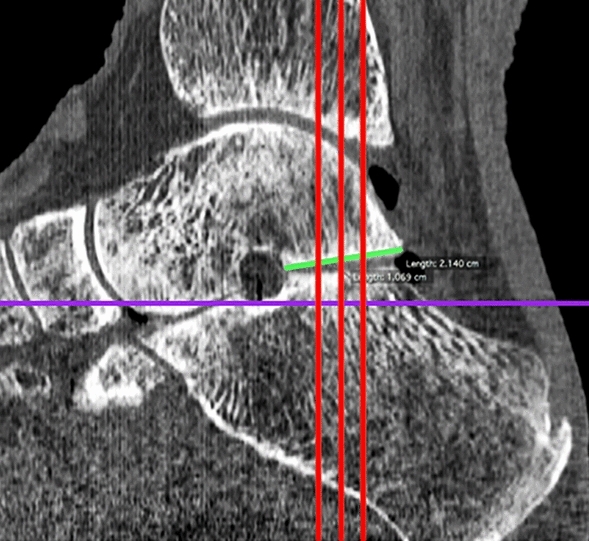
Sagittal CT view demonstrating the definition of the three coronal planes—anterior, middle, and posterior—used for measurement of subtalar vertical angle (SVA) and talar subluxation

Measurements of hindfoot alignment, talar subluxation, coronal posterior facet angle, as well as lateral and anteroposterior (AP) talocalcaneal (Kite) angles were performed on each CT scan [14]. Hindfoot alignment was determined by measuring the subtalar vertical angle (SVA) at three coronal planes along the posterior facet: anterior, middle and posterior, as described by Colin et al. [15, 16]. These three planes were used for measurements of the talar subluxation too [17]. The standard three CT scan reformats (axial, coronal and sagittal) were used for measurements. The sagittal reformat was rotated until it was parallel to the long axis of the talus. In this position, the length of the posterior facet of the talus was measured and the center was marked with a vertical line. Five millimeters anterior and five millimeters posterior to this midline marked the anterior and posterior coronal slices, respectively. Using the tracking feature of Osirix image analysis software (Pixmeo, Geneva, Switzerland), these three planes were identified on the coronal reformats, where the measurements were made (Fig. 4).

Increasing values of the coronal posterior facet angle and SVA indicate an increasing varus deformity. Increasing values of talar subluxation indicate medialization of the talus. Increasing values of the Kite angle in lateral view represent an increasing plantar flexion of the talus. Increasing values of the Kite angle in AP view represent an increasing hindfoot valgus/forefoot abduction. All those increasing values of the outcomes would indicate collapse/instability.

Statistical analysis was performed using SPSS software package (Version 27, IBM SPSS, Armonk, NY, USA). Normality of data distribution was checked with Shapiro–Wilk test, followed by non-parametric evaluation of repeated measures outcomes by Friedman’s and Mann–Whitney tests with Bonferroni correction for multiple comparisons. Level of significance was set at *p* = 0.05 for all statistical tests.

## Results

**Fig. 5:**
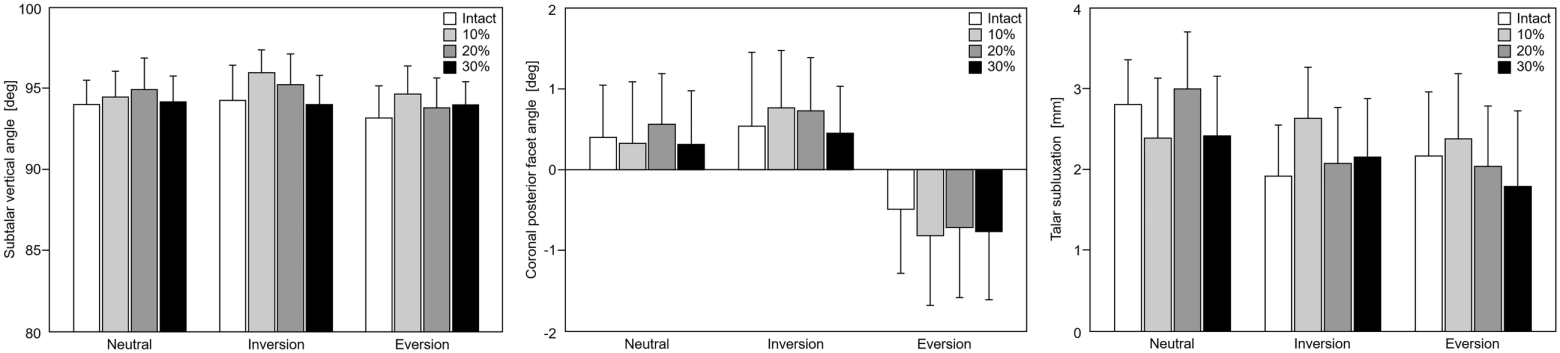
(Left) Bar diagrams depicting subtalar vertical angle, (middle) coronal posterior facet angle and (right) talar subluxation in terms of mean and standard error of mean for neutral, inversion and eversion position of the foot under single-legged stance loading (700 N). No significant differences were detected for each of the outcomes between resected and intact states in same foot positioning

**Fig. 6:**
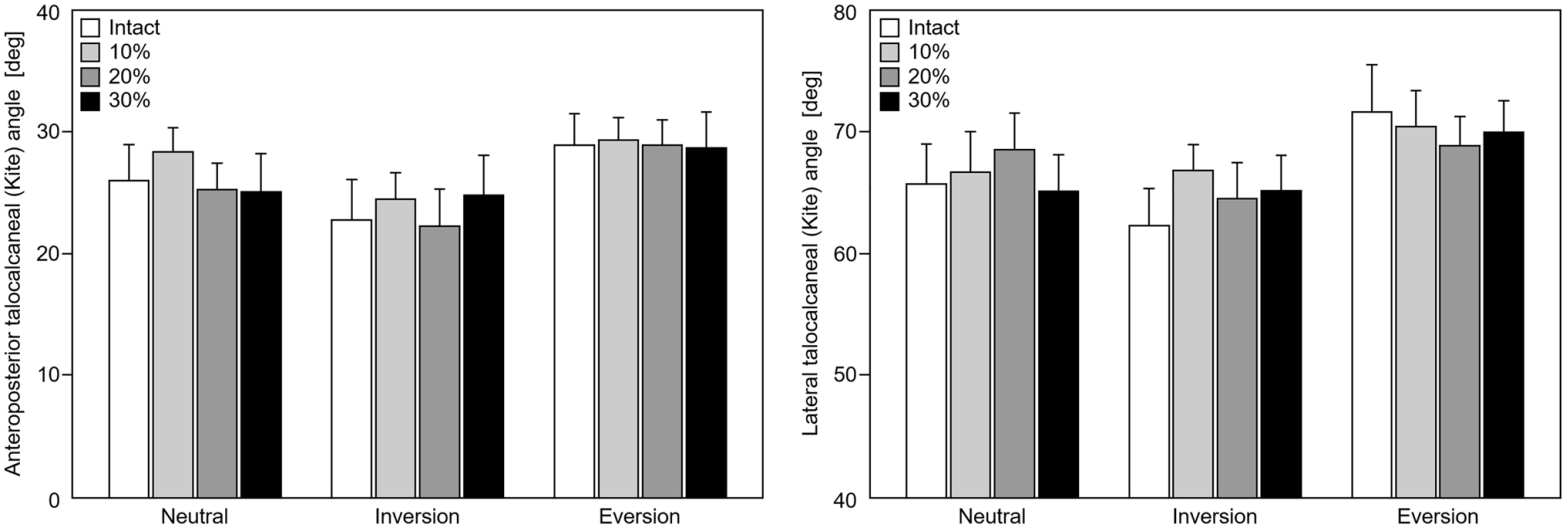
(Left) Bar diagrams depicting anteroposterior and (right) lateral Kite angles in terms of mean and standard error of mean for neutral, inversion and eversion position of the foot under single-legged stance loading (700 N). No significant differences were detected for each of the outcomes between resected and intact states in same foot positioning

Results from the measurements of the SVA and talar subluxation in the middle coronal plane, as well as of the coronal posterior facet angle and the Kite angles in AP and lateral views in the different foot positions (neutral, inversion, eversion) and states (intact, resected) under single-legged stance loading (700 N) are presented in Figs. 5 and 6.

Gross clinical instability was not observed in any of the specimens and any of the loading conditions, foot positions, intact and resected states. No significant differences were detected between resected and intact states (*P* ≥ 0.10), as well as among the resected states in each foot position (*P* ≥ 0.11; Table 1).

**Table 1:** *P* values for comparisons of the outcomes subtalar vertical angle (SVA), coronal posterior facet angle (PFA), talar subluxation (TS), anteroposterior (KA—AP) and lateral (KA—lateral) Kite angles between resected and intact states, as well as among resected states in neutral (N), inversion (Inv) and eversion (Ev) foot positions under single-legged stance loading (700 N). Comparisons for SVA and TS include all measurements at the three coronal planes (anterior, middle and posterior) along the posterior facet

Outcome	Position	Resection versus intact			Among resections
		10%	20%	30%	
SVA	N	≥ 0.48	≥ 0.26	≥ 0.67	≥ 0.69
	Inv	≥ 0.10	≥ 0.12	≥ 0.16	≥ 0.14
	Ev	≥ 0.26	≥ 0.60	≥ 0.58	≥ 0.45
PFA	N	0.62	0.69	0.26	0.14
	Inv	0.29	0.40	0.44	0.75
	Ev	0.87	0.23	0.92	0.39
TS	N	≥ 0.40	≥ 0.58	≥ 0.48	≥ 0.37
	Inv	≥ 0.10	≥ 0.17	≥ 0.17	≥ 0.62
	Ev	≥ 0.87	≥ 0.89	≥ 0.40	≥ 0.77
KA—AP	N	0.40	0.67	0.78	0.11
	Inv	0.40	0.89	0.48	0.23
	Ev	0.48	0.67	0.89	0.69
KA—lateral	N	0.67	0.12	0.48	0.16
	Inv	0.22	0.26	0.13	0.34
	Ev	0.99	0.33	0.67	0.42

## Discussion

Our hypothesis that up to 30% resection of the medial talar facet and the anteromedial portion of the posterior talar facet does not result in biomechanical hindfoot instability could be confirmed.

According to clinical experience, there are two situations where resection of the medial talar facets might lead to clinical improvement—*talocalcaneal tarsal coalition and comminuted medial talar body fractures.*

When nonoperative treatment of a symptomatic *talocalcaneal coalition* fails, operative treatment must be considered [8]. However, there is no consensus regarding the decision when to perform resection or arthrodesis [18–20].

Several factors, including size of the coalition, patient age, neuromuscular function and pre-existing arthritic changes influence this decision [21]. Arthrodesis is a definitive treatment; however, it is not performed without complications. Some surgeons choose arthrodesis over concerns of instability when large resections are necessary or advanced arthrosis is visible [22].

Resection of the medial talus has been reported and more extensively investigated in the setting of *talocalcaneal coalition*. Wilde *et al* treated 20 feet in 17 children less than 16 years old with symptomatic *talocalcaneal coalition* by resection of the coalition bar [21]. In patients with 50% or less coalition of the posterior calcaneal facet area—measured preoperative in coronal CT slices—good or excellent long-term results were reported [21]. In case of preoperative CTs demonstrating a coalition area of more than 50%, fair to poor functional results were communicated, with symptomatic relief but also with a flexible or rigid planovalgus foot, spasm of the peroneal muscles, and radiographic evidence of hindfoot valgus. It was indicated that a coalition of 50% or less was associated with a heel valgus of less than 16° and no signs of arthritis, whereas coalitions over 50% were associated with a heel valgus of more than 16° and radiographic narrowing of the posterior talocalcaneal joint. As a result, resection was proposed as a good treatment for patients with a coalition area of 50% or less, and a heel valgus of 16° or less. For patients with a coalition over 50% and a heel valgus of more than 16° arthrodesis was recommended. To avoid instability, symptomatic medial facet coalition can be treated with subtalar fusion [23]. However, although this procedure reliably reduces pain in the subtalar joint and corrects hindfoot deformity, it restricts inversion and eversion of the hindfoot, so that the remaining intact foot and ankle joints must then accommodate the limited mobility [24]. This can also lead to early arthritis in these neighboring joints, termed as 'adjacent joint disease'. Fusion also carries the risk of nonunion or malunion—complications not being inherent to a simple resection.

Recent approaches combine the resection of the middle facets with interposition of tissue as described by Tower *et al* [6]. They reported a series of four cases in three adolescent patients with resected talocalcaneal middle facet coalition and hyaline cartilage grafting during the same operation. Good clinical outcomes were reported with decreased pain and improved subtalar joint motion, however, subtalar stability was not addressed. Nevertheless, the authors did not specify the amount or quantity of the resected bony tissue.

This presented study demonstrates that the excision of up to 30% of the medial facet and the anteromedial portion of the posterior facet of the talus does not result in significant instability of the subtalar joint and may advocate for a more aggressive resection of *talocalcaneal coalitions*. Excision might thus be an option for some patients without concern for hindfoot collapse.

Another observed clinical pathology occurring to the medial talus is the mentioned *comminuted talar body fracture*. For displaced intra-articular fractures, operative management is necessary [3]. However, the inferior portion of medial talar body is not easy to access surgically. Some surgeons attempt closed or percutaneous reduction of the articular surface, while others resect the fragment, particularly with multiple articular fragments that are not amenable to anatomic reduction and stable fixation [3, 10, 25].

The more common lateral talar fractures are already further extensively studied. There are existing in vitro experimental studies addressing subtalar instability after partial excision of the lateral talar process and demonstrating that lateral talar process fractures can be excised without causing instability of the subtalar joint [26, 27].

Based on these studies and the present investigation, it can be concluded that when the ligamentous support remains intact, the resection of even larger portions of the talus is possible without significant changes in the biomechanical stability of the subtalar joint.

This study complements the existing literature by giving evidence that *medial talar body fractures* can also be excised without causing undue instability of the subtalar joint. While clinical correlation is needed, this encourages both open and arthroscopic excision in the setting of fragments intruding on the tarsal tunnel or symptomatic nonunions encompassing up to 30% of the posteromedial facet of the talus.

To our knowledge, this is the first experimental study investigating hindfoot stability after combined resection of the medial facet and the anteromedial portion of the posterior facet. During the operative dissection of the specimens in the current study special attention was paid to maintain the integrity of the interosseous ligament and the deltoid insertion, as well as to avoid instability caused by the missing ligamentous support. This was consistent with previous reports documenting the considerable importance of the talocalcaneal interosseous ligament to subtalar joint stability [28–32]. This ligament complex can be disrupted in the setting of fracture and/or dislocation though, so clinical evidence is needed to support our findings.

The current study has some limitations similar to those inherent to all human cadaveric biomechanical studies. A limited number of specimens were used, thus restricting generalization to actual patients. Static loading and constrained foot positions do not replicate the complexities of dynamic human gait. Without the dynamic stabilizers of the tendons surrounding the hindfoot, however, this allows to test the true structural importance of the posterior medial facet. In addition, the specimens were not tested to failure and all tests were performed in the same setting to avoid excessive tissue fatigue. Retrospectively, an extension of resection up to significant subtalar joint instability would have been desirable.

Further, it is possible that subtle instability was not detected with the current test design and measurement techniques, or that weight-bearing on a partially resected talus could have attenuated the remaining ligaments and lead to arthrosis or instability over time. Despite these limitations, one of the benefits of the current model was the ability to directly compare the biomechanical behavior of an intact specimen versus its performance following sequential resections in vitro, thus allowing to account for anatomic variation and alignments among the specimens something difficult to accomplish in an in vivo setting.

## Conclusion

In a biomechanical setting, resecting up to 30% of the medial facet and anteromedial portion of the posterior facet based on the intact talus width does not result in any measurable instability of the subtalar joint in presence of intact ligamentous structures. This could be particularly relevant in the treatment of *talocalcaneal tarsal coalitions* and *comminuted medial talar body fractures*.

## Data Availability

The datasets used and/or analyzed during the current study are available from the corresponding author on reasonable request.
